# Entropy transfer from solar radio bursts to energetic particles

**DOI:** 10.1126/sciadv.adz7419

**Published:** 2025-11-26

**Authors:** George Livadiotis, Manuel E. Cuesta, Leng Y. Khoo, Mitchell M. Shen, David J. McComas, Marc Pulupa, Stuart D. Bale, Roberto Livi

**Affiliations:** ^1^ Department of Astrophysical Sciences, Princeton University, Princeton, NJ 08544, USA.; ^2^ University of California, Berkeley, CA 94720, USA.

## Abstract

Space plasma thermodynamics is thought to be affected by wave activity. Here, we show that solar radio bursts (SRBs) can transfer entropy to solar energetic protons (SEPs), affecting their thermodynamics. In particular, our analysis (i) detects the statistically significant SEP density fluctuations, associated with SRB activity that triggers a systematic increase in the thermodynamic kappa; (ii) estimates the polytropic index of SEPs, which is anticorrelated with kappa, serving as an independent measure to validate the increase in kappa; (iii) derives the entropy transfer by using its theoretical relationship with kappa; and (iv) compares SRB wave intensity with the entropy transferring to SEPs to demonstrate their wave-particle coupling. We lastly expose the thermodynamic association between type III SRB wave intensity and SEP entropy transfer as well as their respective coupling, thus developing a paradigm for further systematic investigations among other types of wave activity and particle populations.

## Teaser

Entropy transfers from SRB activity to SEPs, providing a unique way to measure their wave-particle coupling.

## INTRODUCTION

Space plasmas are typically collisionless particle systems; their particle-particle collisions are rare, and thus, thermalization is often carried out by wave-particle interactions (*1*, *2*). The plasma waves in collisionless space plasmas behave as collisions in collisional systems. Charged particles interact with electromagnetic waves without physical collisions, resulting in the transfer of momentum and energy among particles through wave-particle interactions, thus playing the role of physical collisions, through which the plasma relaxes toward thermal equilibrium [e.g., (*1*, *3*–*5*)].

Collisionless space plasmas have fundamentally different thermodynamics than particle populations in collisional plasmas or gases. Highly collisional particle systems are described by classical thermodynamics, which is based on the absence of correlations among particle energies. This assumption was considered even in the original foundations of thermodynamics through its connection with statistical mechanics (*6*). According to this, a particle system with no correlations resides in the classical thermal equilibrium, where the particle velocities (and kinetic energies) of systems are described by the Maxwell-Boltzmann (MB) distribution.

Particle correlations are largely eliminated when collisions occur at a frequency *f*_col_ higher than the characteristic frequency of correlations, that is, the plasma oscillation frequency *f*_pl_ (i.e., *f*_col_ ⪆ *f*_pl_), or equivalently, when the mean free path, *L*_mfp_ ~ θ/*f*_pl_, is smaller than the Debye length λ_D_ ~ θ/*f*_pl_ (i.e., *L*_mfp_ ⪅ λ_D_) (θ is the proton thermal speed); on the contrary, in collisionless particle systems, the collision frequency between charged particles is smaller than the plasma frequency, or equivalently, the mean free path is larger than the Debye length (*1*, *7*, *8*). Therefore, collisional plasmas are well described by classical thermodynamics, because the particle-particle collisions destroy the particle correlations. In contrast, collisionless space plasmas have particle correlations that prevail over collisions and thus cannot be described by MB distributions and classical thermal equilibrium.

**Fig. 1.**
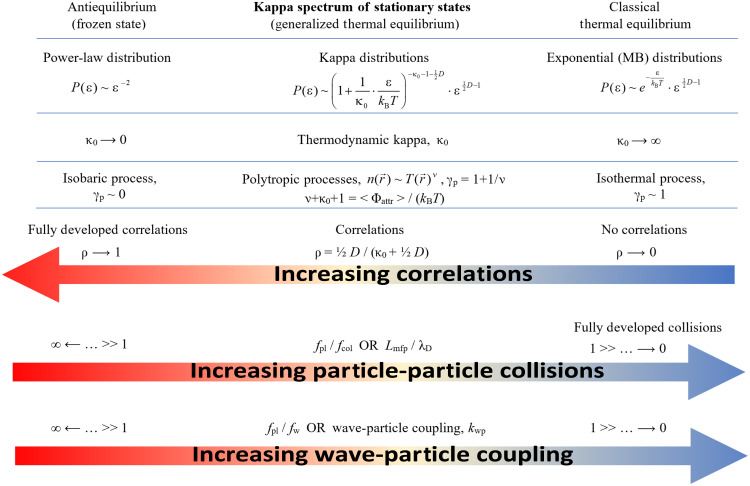
Spectrum of thermodynamic stationary states. This constitutes the generalized thermal equilibria described by kappa distributions, parameterized by the thermodynamic kappa κ or its invariant, dimensionality-independent value, κ_0_ = κ − ^1^/_2_*D*. The extremes are the classical equilibrium at κ_0_ ⟶ ∞ and the antiequilibrium (or frozen state) at κ_0_ ⟶ 0, where the distribution degenerates to the MB exponential and a power law, respectively. Kappa is connected with the polytropic processes, characterized by the polytropic index γ_p_ (or ν) and the correlation coefficient ρ (measure of correlations). In general, collisions are competing with correlations to define the stationary state that the plasma is stabilized in. Particle-particle collisions and wave-particle interactions have this same effect, to increase the kappa, and thus, the entropy, of the particle system. While physical collisions are known to be quantified by comparing collision with plasma frequencies, wave-particle interactions can be quantified by comparing wave and plasma frequencies or equivalently through the wave-particle coupling *k*_wp_, as shown here.

In collisionless plasmas, particle correlations can be generated by long-range interactions, and the absence of (or at least, the existence of less frequent) collisions allows these correlations to survive, leaving the system to reside in stationary states described by kappa distributions—called generalized thermal equilibrium (*9*–*18*). This contrasts with the classical thermal equilibrium, which is a limiting version of thermal equilibrium described by MB distributions. Common processes that induce correlations in collisionless space plasmas are Debye shielding and magnetic coupling, which play important roles in the generation of kappa distributions in plasmas (*16*). Occasional wave-particle interactions induce disorder into thermodynamics, shifting the stationary state that the system is residing in, from a more organized state toward a less organized one, characterized by lower particle correlations (Fig. 1). The coupling between wave activity and particles can provide a measure of their impact on the particles’ thermodynamics. This paper measures this coupling constant and shows a robust method for its derivation, thereby enhancing its physical meaning and importance.

The parameter kappa (κ) that labels and shapes the kappa distributions can be thermodynamically defined through the correlations between particles. Correlations induce order in the involved systems and reduce their entropy, thus producing a missing entropy, called the entropy defect, whose normalized percentage defines and measures the magnitude of the defect, 1/κ (*11*, *19*). Inverse kappa measures the correlations of particle kinetic energy per degrees of freedom [e.g., (*9*, *13*)]; this is in parallel to the other intensive parameter, the temperature, which measures the mean particle kinetic energy per degrees of freedom (*20*, *21*). Temperature and kappa are two independent thermodynamic parameters having unique kinetic and thermodynamic definitions (*21*). While temperature varies with transferring of energy (heating), kappa varies with transferring of entropy (*19*).

As described above, classical thermal equilibrium constitutes a limiting stationary state, which is given by the kappa distribution with κ ⟶ ∞, recovering MB distributions (*14*, *20*), indicating that correlations approach zero at this limit. Space plasma thermodynamics is characterized by a spectrum-like arrangement of the values of kappa (correlations), where one limit leads to MB distributions and the other limit leads to the power-law behavior of “antiequilibrium” (*9*, *14*). Each thermodynamic stationary state that a space plasma resides in is characterized by a certain value of kappa κ or, equivalently, a correlation coefficient ρ, or a polytropic index γ_p_, where each of these parameters characterizes the thermodynamic processes—called “polytropes” (see Fig. 1) [e.g., (*22*); for other similar measures, see (*23*)].

Recent developments regarding the thermodynamic properties of solar energetic protons (SEPs) have opened an area of research that may help our understanding of SEP acceleration and their connection across a variety of solar events, such as solar flares and coronal mass ejections (CMEs) (*24*, *25*). SEP acceleration mechanisms, such as diffusion shock acceleration (*26*–*30*), affect the SEP particle velocity distributions depending on the availability of seed particles usually with suprathermal energies generally between ~10 keV and ~1 MeV per nucleon (*31*). When particles are accelerated by wave-particle interactions, magnetic interactions, or other coherent structures, they can eventually stabilize into thermodynamic stationary states different than their original one.

Wave activity is specifically expected to influence thermodynamics of SEPs, and a characteristic example of solar wave activity is a solar radio burst (SRB). SRBs are intense, transient radio emissions generated by various plasma processes driven by energetic events, primarily solar flares and CMEs (*32*, *33*). These radio bursts span a broad frequency range and are classified into five main types (I to V) according to their spatial and temporal characteristics. Among them, type III SRBs are most frequently observed through spectral signatures of rapid drifting from high to low frequencies, often corresponding to solar flares (*34*–*36*). They are associated with suprathermal electrons that are accelerated and propagated along open field lines through the solar corona and heliosphere. As these electron beams travel outward, they produce Langmuir waves in regions of decreasing plasma intensities, subsequently producing plasma wave emission near the local plasma frequency $f_{\text{pl}}\propto \sqrt{n_{e}}$ , where *n_e_* is the solar wind electron density. Hence, type III SRBs offer valuable diagnostics of both particle acceleration processes and local plasma conditions. Solar energetic electrons (SEEs) are also known to be accelerated to higher energies through eruptive events such as flares and CMEs (*37*, *38*). In situ observations commonly show that energetic electron events are almost invariably accompanied by type III SRBs, highlighting a close association between electron acceleration and radio wave generation (*39*, *40*).

In the near-sun solar wind, there are various type III SRB–related studies that investigate wave emissions (*41*) and their connection to energetic particles (*42*) observed by Parker Solar Probe (PSP). Large SEP events are known to be associated with complex type III bursts (*43*, *44*); these bursts have a long duration (>15 min) and occur at low frequencies (<14 MHz) (*45*).

However, to this date:

1) No thermodynamic association between type III SRB wave activity and SEPs has ever been reported. The frequency of type III SRBs typically lies higher than the local characteristic plasma, as they are generated at the frequency of the local plasma of the distant source close to the Sun (where the local plasma frequency is higher) (*36*). In the absence of resonance between the radiation and plasma particle frequencies, no heat can be efficiently transferred from the radiation to the plasma protons. However, the difference in frequencies does not prevent disorder caused by wave activity from affecting the SEP thermodynamics (*46*). Therefore, while energy cannot be effectively transferred from type III SRBs to SEPs, leaving their temperature unaffected, transfer of entropy can occur, quantifying the added disorder and affecting the SEP thermodynamic kappa.

2) No entropy transfer from wave activity to particles has ever been measured. As classical thermodynamics is not applicable to space plasmas, the entropy associated with the statistical framework of kappa distributions must be used instead. The entropy transfer is one-to-one related to the increase/decrease in thermodynamic kappa. The application of the kappa-tail technique on energy-flux particle spectra enables us to estimate the kappa time series (*24*) and, thus, to infer the entropy transfer (*19*).

3) No measurement has ever been made of the wave-particle coupling through entropy transferring. A systematic investigation along these lines of research will establish a consistent characterization of the wave-particle coupling and its potential variations over different types of wave activities and particle populations.

The effect of type III SRBs on SEP thermodynamics is ideal for investigating the connection to wave-particle interactions, because these SRBs can transfer entropy rather than energy, affecting the SEP thermodynamics in a way different than acceleration or heat. The purpose of this paper is to present an example of wave-particle interactions, where the wave activity of type III SRBs transfers entropy to SEPs, affecting their thermodynamics. In addition, we provide a solid framework to study this effect and the wave-particle coupling between the SRB wave activity and the SEP thermodynamics, more generally.

## RESULTS

### Thermodynamic processes of SEPs affected by SRBs

**Fig. 2.**
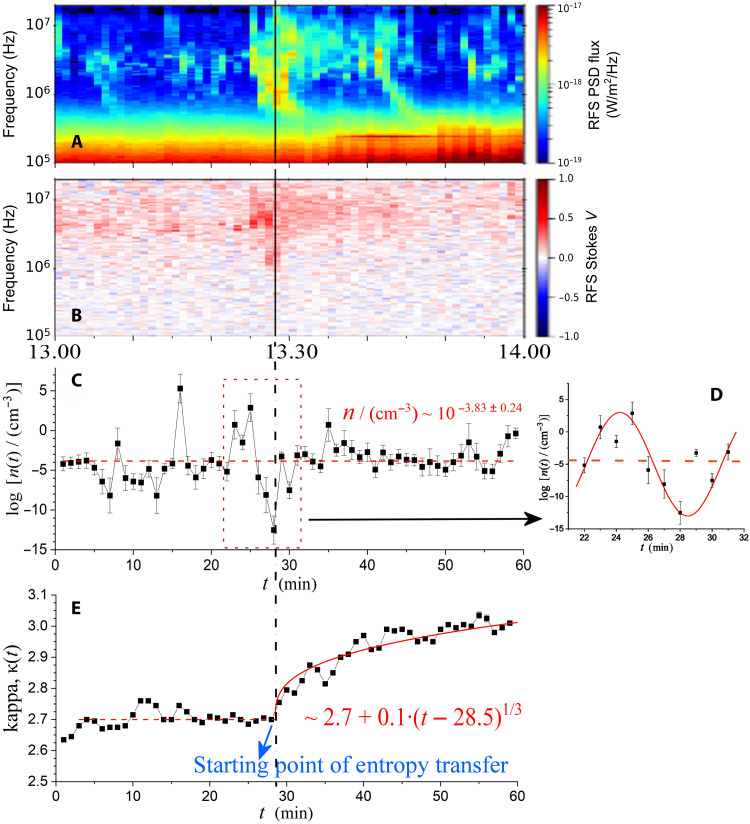
Comparison between measurements. (**A**) Spectrogram showing the wave activity with the intensity [power-spectrum density (PSD) flux] plotted on a log scale. (**B**) Polarization analysis of the wave emissions (Stokes parameter *V*), (**C**) density of SEPs derived from sequential SEP spectra using the kappa-tail technique (see Materials and Methods and Supplementary Text S1) (*24*), and (**D**) detailed SEP density profile showing the sinusoidal-like fluctuation from *t* ~ 23 min to *t* ~ 32 min. (**E**) Thermodynamic kappa of SEPs.

The SRB wave activity interferes with the particle correlations, causing disorder to spread in the particle system and disturbing its thermodynamics (Fig. 2). The effect of this disorder on thermodynamics is measured by increasing the effective degrees of freedom Δ*D* or the entropy Δ*S* of the system. The increase in the thermodynamic kappa is connected to both the increases in the degrees of freedom, $\Delta \kappa =\frac{1}{2}\Delta D$ (*9*), and in the entropy $\Delta \left(1/\kappa \right)=\left(2/S_{\infty }^{2}\right)\cdot \Delta S$ , with *S*_∞_ indicating the entropy for κ ⟶ ∞ (*19*).

The process of entropy transfer is a nonstationary thermodynamic state, but it can be approached by a series of local stationary microprocesses. This follows the local thermodynamic equilibrium (LTE) approach, where nonequilibrium state processes can break down into a connection of local thermodynamic equilibria [e.g., (*47*)]. LTE is realistic as long as the transfer of disorder is slow enough for the disturbed system to have time to reach local stationarity (local kappa distribution and polytropic behavior). Each local stationary state is characterized by a certain value of kappa (and polytropic index, satisfying Eq. 10), where the connection of all local stationary states into the nonstationary leads to the time-dependent kappa.

At times before the SEP thermodynamics is disturbed, the thermodynamic kappa is nearly constant, κ ~ 2.7, while the respective polytropic index is ν ~ −2.2; thus, the kappa-polytropic relationship holds, that is, κ *+* ν ~ ^1^/_2_. The density and temperature are statistically constant (i.e., the fitting of their values by a constant has a significant statistical confidence—*P* value >0.05), having average values, $\text{log}\left(n/{\text{cm}}^{-3}\right)≅-3.83\pm 0.24$ and log(T/keV)≅2.86±0.08 [corresponding to $n≅\left(1.5\pm 0.8\right)\times 10^{-4}\;{\text{cm}}^{-3}$ and T≅(0.72±0.13) MeV ], respectively (Fig. 2C). The instantaneous values of density and temperature fluctuate around these averages. Their fluctuations are not random but determined by a single polytropic process and characterized by the polytropic index ν ~ −2.2.

There were several distinct SRB emissions recorded within the examined period, that is, between the hours of 13:00 UTC and 14:00 UTC of 16 February 2022 (see the “Observational data” section). The strongest type III SRB was observed around approximately 13:25 UTC, with some polarization features appearing earlier near approximately 13:22 UTC, peaked at approximately 13:28.5 UTC, and expanded up to approximately 13:30 UTC. Then, another type III SRB was observed from approximately 13:41 to approximately 13:49 UTC, as well as a suspicious type IV SRB in between these two main SRBs. Also, there is spared SRB wave activity before the main one.

The SRB wave activity (Fig. 2, A and B) is linked to sinusoidal perturbations in density, with the main type III SRB between approximately 13:22 and approximately 13:30 UTC linked to the density perturbation shown in the magnifying panel (Fig. 2D), which also takes place within the same period of time. This large fluctuation affects the statistics of density values (Fig. 2C) so that they cannot be well fit by a constant (see the “Thermodynamics of SEPs” section and Supplementary Text S2). The sinusoidal perturbation in density appears near the trigger time, *t*_0_ ~ 13:28.5 UTC, where the kappa starts to have a coherent, or statistically significant, increase (Fig. 2E). At this time, the SEP system becomes nonstationary but remains locally stationary according to LTE, namely, it is described by a time-dependent thermodynamic kappa κ(*t*) and polytropic index ν(*t*).

**Fig. 3.**
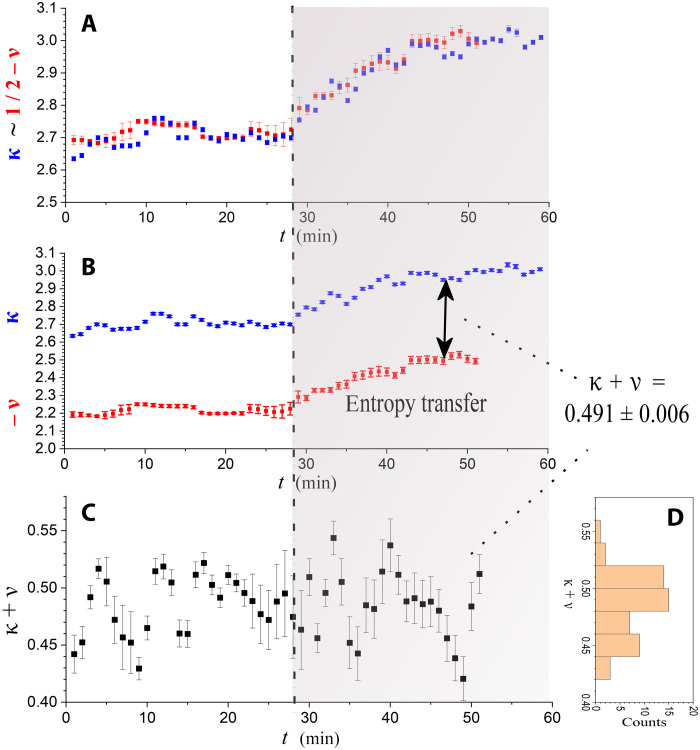
Relationship between the thermodynamic kappa and the polytropic index. (**A**) Plot of the values of κ and ^1^/_2_ − ν, showing their coincidence. (**B**) Plot of the values of κ and −ν, showing their approximately constant difference, although they were estimated by different and independent methods. The weighted mean of their differences is 0.491 ± 0.006. The values of κ + ν are plotted in (**C**) and their histogram in (**D**). (Polytropic indices were not measured for *t* > 53 min.)

The polytropic index exhibits similar behavior. At the trigger time, it starts decreasing by the same amount that kappa increases, i.e., Δν(t)≅−Δκ(t) ; thus, their sum remains constant (at ~^1^/_2_). Specifically, the weighted average of all the values of (κ*_i_* + ν*_i_*) is estimated to be 0.491 ± 0.006. Figure 3A plots κ*_i_* and ^1^/_2_ − ν*_i_*, where we observe that they share the same trend. Figure 3B highlights the constant difference (κ*_i_* + ν*_i_*) between κ*_i_* and −ν*_i_*. Figure 3C presents the time series of their sum, (κ*_i_* + ν*_i_*), while their respective histogram, *P*(κ + ν), is shown in Fig. 3D. We stress the fact that the thermodynamic kappa and the polytropic index are estimated using different and independent methods: For the thermodynamic kappa, we use the kappa-tail technique that estimates the weighted mean of the spectral indices for two sequential spectra (“Thermodynamics of SEPs” section), while for the polytropic index, we use the thermodynamic parameter method that analyzes the density and temperature logarithm profile and fits a linear relationship (“Polytropic processes of SEPs” section). Therefore, the result that the values of the kappa and polytropic index match their theoretical expression (shown in Eq. 10) is a strong support of the theory of kappa distributions and their connection to polytropes. This validates the observed increase in kappa, and thus, it can be further used for determining the entropy transfer.

Last, we note that matching the relationship between the polytropic index and the coherent variability of kappa verifies that the effect on SEP thermodynamics is local and not transported. Nonlocal changes of kappa, e.g., because of a modification at the source (the shock) that was transported to PSP’s location, would persist over larger timescales than the period observed after the trigger time, and then, the thermodynamics of the particles would change stochastically along the path of their transportation and/or through its interaction with the magnetic field (*48*). Moreover, transport changes do not match the polytropic behavior. In particular, the variability of density and temperature reveals the local polytropic process and not the transported thermodynamic variability, as the relationship between the thermodynamic kappa and the polytropic index is a result of local thermodynamics. Therefore, the verification of the locality of the coherent variation of kappa, through its matching with the polytropic index relationship, is a requirement of the applicability of the method.

### Entropy transfer to SEPs

In particular, the SEPs receive “disorder,” corresponding to increasing effective degrees of freedom Δ*D*(*t*) and entropy ΔS(t) , determined by the increase in the thermodynamic kappa Δκ(t) , as follows

$$\Delta D\left(t\right)=2\cdot \Delta \kappa \left(t\right),\Delta S\left(t\right)=\frac{1}{2}S_{\infty }^{2}\cdot \left[\frac{1}{\kappa \left(t_{0}\right)}-\frac{1}{\kappa \left(t_{0}\right)+\Delta \kappa \left(t\right)}\right]$$ (1)

where $S_{\infty }$ denotes the classical Boltzmann Gibbs adaptation of entropy of the system, that is, for κ ⟶ ∞; all entropies are expressed in units of Boltzmann constant, *k*_B_. The thermodynamic kappa fits the empirical model $\kappa \left(t_{0}\right)≅2.7$ , $\Delta \kappa \left(t\right)≅\frac{1}{2}A\cdot {\left(t-t_{0}\right)}^{\alpha }$ , with *A* = 0.2 and α = 1/3.

We model the relationship between the normalized entropy rate and the normalized spectral intensity, as follows. The entropy rate is normalized using the function (dΔS/dt)/ΔS and the characteristic time lag τ, measured from the start of the wave activity to the start of the effect on SEP thermodynamics (trigger time), namely, τ⋅dln(ΔS)/dt . On the other hand, the spectral intensity is normalized by its value at the trigger time, $J_{0}\equiv J\left(t_{0}\right)$ , i.e., $J/J_{0}$ . Therefore, their connection can be expressed by some coupling function *f* so that $\tau \cdot d\text{ln}\left(\Delta S\right)/\mathrm{dt}=k_{\text{wp}}\cdot f\left(J/J_{0}\right)$ , where $k_{\text{wp}}$ stands for the wave-particle coupling between the cause (intensity of wave activity) and the effect (transferred entropy on SEP thermodynamics). The coupling function’s exact form is not actually needed here, as it is approached by a power-law expansion (for $J/J_{0}$ < 1), that is, $d\text{ln}\left(\Delta S\right)/\mathrm{dt}\propto {\left(J/J_{0}\right)}^{p}$ , for some intensity exponent *p*. Small intensities do not appear to be linked with a significant increase in kappa, and thus, do not initiate a significant entropy transfer, so we expect the function to be nonlinear with the exponent *p* > 1, which must be near the next order term, i.e., *p* ~ 2. Then, the complete relationship between entropy transfer and wave intensity is written as

$$\frac{d\text{ln}\left(\Delta S/k_{B}\right)}{\mathrm{dt}}≅k_{\text{wp}}\cdot \frac{1}{2}\cdot \frac{1}{\tau }\cdot {\left(\frac{J}{J_{0}}\right)}^{2}+O{\left(\frac{J}{J_{0}}\right)}^{3}$$ (2)

The characteristic time τ scales with frequency as $\tau \left(f_{w}\right)=\tau \left(f_{\text{pl}}\right)\cdot {\left(f_{w}/f_{\text{pl}}\right)}^{-\alpha_{d}}$ (*49*) with exponent $\alpha_{d}≅0.66\pm 0.03$ , where $\tau_{\text{pl}}\equiv \tau \left(f_{\text{pl}}\right)$ is the time for restoring equilibrium. The latter can be estimated by the time lag τ, measured from the start of the wave activity of type III SRB at 13:25 UTC to the start of the thermodynamic effect on SEPs at 13:28.5 UTC, i.e., $\tau_{\text{pl}}≅3.5\pm 0.5\;\text{min}$ . The characteristic time is involved in Eq. 2 at the trigger time, i.e., $\tau \left(f_{w0}\right)=\tau_{\text{pl}}\cdot {\left(f_{w0}/f_{\text{pl}}\right)}^{-\alpha_{d}}$ ; therefore, the coupling constant is expressed by

$$k_{\text{wp}}≅\text{slope}\cdot 2\tau_{\text{pl}}\cdot {\left({\langle f_{w}\rangle }_{h,0}/f_{\text{pl}}\right)}^{-\alpha_{d}}$$ (3)

We included the harmonic average ${\langle f_{w}\rangle }_{h,0}\equiv {{\langle {f_{w}}^{-1}\rangle }_{0}}^{-1}$ , calculated over all the frequencies at the trigger time; we find ${\langle f_{w}\rangle }_{h,0}≅3.7\pm 0.4\;\left(2.8\right)\;\text{MHz}$ . For an electron plasma density $n_{e}≅100\;{\text{cm}}^{-3}$ , the plasma frequency is $f_{\text{pl}}≅0.09\;\text{MHz}$; thus, ${\langle f_{w}\rangle }_{h,0}/f_{\text{pl}}≅41\pm 4\;\left(30\right)$. In the “Measuring the entropic transfer” section, we show that SRB wave activity and entropy transfer to SEPs follow the relationship in Eq. 2; the respective statistical analysis estimates the slope $≅0.0673\pm 0.0012\;\left(0.0062\right)\;{\text{min}}^{-1}$ . Hence, $k_{\text{wp}}≅0.041\pm 0.009$.

### Causality timeline: From SRB wave activity to SEPs

**Fig. 4.**
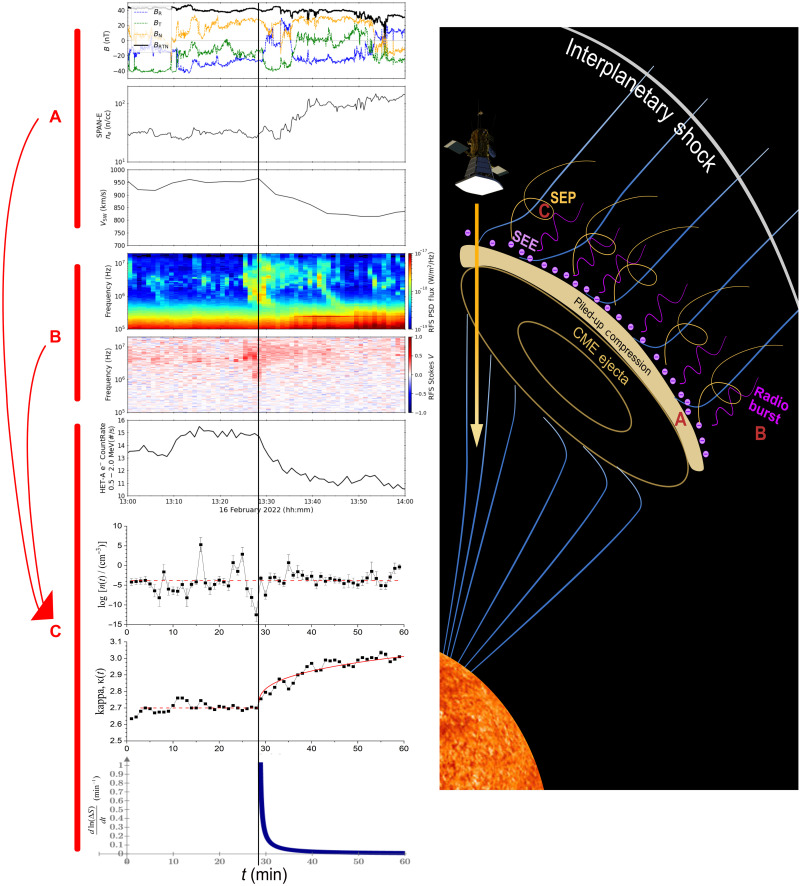
Causality diagram along PSP’s trajectory. We plot PSP’s trajectory through the outward moving ICME (right) along with the following diagrams (left): (**A**) in situ–measured parameters of the magnetic field, solar wind electron density, and solar wind proton speed; (**B**) power spectral density of wave emissions and polarization analysis of the wave emissions; and (**C**) measured HET-A electron count rate, derived SEP density, thermodynamic kappa, and entropy transfer rate (in descending order). The vertical black line marks the time at which the thermodynamic kappa begins to increase, indicating the start of significant entropy transfer. (Details on the right panel: Blue lines indicate the magnetic field, the yellow arrow indicates the trajectory of PSP during this event, and pink circles represent the energetic electrons measured by PSP/HET before PSP enters the PUC region, indicated by yellow block and followed by the CME ejecta.)

PSP observed an interplanetary shock around 07:25 UTC on 16 February 2022 (*50*). The period of interest (13:00 to 14:00 UTC, 16 February 2022) happened inside the interplanetary CME (ICME) sheath. Figure 4 summarizes PSP’s observation during the event.

During the period of interest, we observe an increase in SEP electrons around ~1 MeV by both HET-A (A-side of the high-energy telescope) and LET-A (A-side of the low-energy telescope) channels on the Integrated Science Investigation of the Sun (IS⊙IS), implying a local acceleration of SEEs from 13:10 to 13:30 UTC. Meanwhile, the solar wind density is increasing and the velocity is decreasing, as the PSP spacecraft enters the piled-up compression (PUC) region (Fig. 4). This turbulent region of enhanced plasma density and magnetic field strength is formed within the sheath and ahead of the ICME because of its large expansion rate. In general, PUC regions act as dynamic boundaries in the solar wind, often rich in turbulence and wave activity (*50*–*52*).

At approximately 13:22 UTC, PSP observed a significant type III emission, consistent with the enhancement of SEEs and the sinusoidal disturbance in the SEP density; SRBs are generated at the frequency of the local plasma, in this case, at a distant source close to the Sun where the local plasma frequency there is higher than at the measurement point. Before this, no statistically significant thermodynamic coupling had been observed between typical SRB wave activity and SEPs.

Both type II and type III SRBs interact with the nonlinear environment upstream the PUC region, potentially exciting secondary wave generation and plasma instabilities. Fine structures and spectral evolution of type III bursts are often linked to plasma inhomogeneities like near the PUC (*53*). Moreover, these secondary waves and instabilities can scatter and/or isotropize the distribution of SEEs, effectively reducing their excess upstream the PUC.

The energy carried by type III SRBs is orders of magnitude lower than what is needed for significantly heating and altering the kinetic energy of protons. In addition, protons do not resonate with the frequencies of type III bursts. Without resonance, there is no efficient mechanism for direct wave-particle energy transferring from the SRBs to energetic protons. Local acceleration driven by type II and type IV SRBs can facilitate energy transfer through magnetic reconnection, turbulence, compressive structures, or interplanetary shocks (*54*, *55*). Even though direct energy transfer from the coincidentally observed type III SRBs is unlikely, type III SRBs can still transfer entropy through these wave-particle interactions to the thermodynamics of the SEP population. There is no need for resonance to achieve entropy transfer (*46*). Entropy transfer often occurs through nonresonant mechanisms that shuffle particles in the velocity space without changing their total energy. For example, ballistic modes and parallel advection can stretch and fold the distribution function, increasing entropy even in the absence of direct energy exchange. In general, entropy can be redistributed via other physical mechanisms, such as (i) an environment with strong nonlinear wave-wave and wave-particle coupling, (ii) phase mixing, (iii) turbulent cascades, and (iv) shear flows and fine-scale structures (*46*, *56*). Moreover, even if SEPs do not absorb energy from the radiation, they can be influenced by fluctuations in the electromagnetic field, which may alter their phase-space distribution. SEPs moving through regions of fluctuating radiation fields may experience stochastic deflections or velocity-space diffusion. These nonlinear interactions can increase entropy by broadening or distorting the distribution function—without necessarily increasing the temperature (*57*). By reshaping the distribution function without affecting the temperature on average, it returns to varying the value of other remaining thermodynamic parameters, that is, increasing kappa, which is one-to-one connected with the increase in entropy (*19*).

Although the connection between SRB wave activity with plasma and SEP thermodynamics is complex, another potential mechanism linking high-frequency SRB wave activity to the entropy of plasma and energetic particles can be purely based on thermodynamic aspects, relying on the weakening of plasma shielding and the resulting correlations. When electromagnetic wave with frequencies higher than the plasma frequency propagates within the plasma, the plasma electrons cannot effectively respond to and shield the rapidly oscillating wave field. The ability of the plasma to neutralize localized electric fields diminishes, thus making charge shielding effectiveness weaker and allowing external influences to penetrate more easily, and therefore, the Debye length λ_D_ expands; as λ_D_ increases, the Debye exponential term of electron screening faints, becoming closer to unity (*7*, *8*). On the other hand, when the Debye length increases under constant density and temperature (on average), it drives the polytropic index γ_p_ to increase, according to Δlog γ_p_ ~ 2*Δlog λ_D_ (*7*); this is equivalent to a decrease in the polytropic index ν (= 1 + 1/γ_p_) or an increase in the thermodynamic kappa (Δκ ~ Δν), and then, the increase in kappa leads to the entropy transfer (*19*). From the point of view of thermodynamics, when the electron shielding weakens, it breaks apart the particle correlations, as these are established among the energies of all the charged particles (e.g., protons and electrons) localized within a Debye sphere. As the correlations diminished, the value of thermodynamic kappa increases (the inverse kappa measures exactly the correlation coefficient; see Fig. 1). Note that both plasma and energetic protons are thermodynamically connected as parts of the overall distribution [e.g., see figure 4 in (*25*)]. Then, the decrease in the “order” induced by correlations leads to the increase in the entropy. This follows the basic concept of thermodynamics, the entropy defect (*11*, *12*).

Therefore, type III SRBs can transfer entropy to plasma and energetic particles through strong nonlinear environments, such the one developed upstream the PUC, by weakening the Debye shielding and generating secondary waves and plasma instabilities. The transferred disorder diminishes the correlations in plasma and energetic particles and thus decreases the “order” induced by correlations, leading to the increase in entropy.

We recap the above interplays into a causality diagram, (A) + (B) ⟶ (C), outlined as follows (Fig. 4):

1) Upstream PUC is a strong nonlinear environment that can affect plasma and energetic particles. Figure 4 plots the in situ–measured parameters of the magnetic field, solar wind electron density, and solar wind proton speed, which are coming along the presence of PUC.

2) The emission of coincidental type III SRBs, whose stronger wave activity starts near approximately 13:22 UTC, followed by a complex secondary wave activity emerging throughout the period of interest past the trigger time. Figure 4 plots the observed power spectral density and polarization analysis of the SRB wave emissions.

3) The nonlinear coupling upstream the PUC and the coincidental passing of type III SRBs provide the necessary conditions for the entropy transfer, which is consistent with the enhancement in SEE density upstream the PUC that reduces upon the effect of SRBs, aligned with the sinusoidal disturbance in SEP density. At the trigger time of approximately 13:28.5 UTC, type III SRBs start the entropy transferring through weakening the Debye shielding and generation of secondary waves and plasma instabilities that also reduce the excess of SEEs. Meanwhile, the thermodynamic kappa of the energetic protons increases at a rate corresponding to the rapid transfer of entropy from waves to energetic protons. Figure 4 plots the measured HET-A electron count rate, derived SEP density, thermodynamic kappa, and entropy transfer rate.

## DISCUSSION

We presented a case study of a wave-particle interaction, where the wave activity of type III SRBs disturbs and transfers entropy to SEPs, affecting their thermodynamics. In particular, a sinusoidal density fluctuation is observed near type III activity, accompanying an increase in thermodynamic kappa of SEPs and, thus, an entropy transfer from the SRB wave activity to the SEPs. This is a consequence of wave-particle interactions, which affects the particle thermodynamics in a way similar to particle-particle collisions and is significant in collisionless space plasmas. The estimated polytropic index decreases by the same amount that the kappa increases, indicating the validation of kappa distributions and their connection with polytropic processes. Matching the relationship between the polytropic index and the coherent variability of kappa verifies that the effect on thermodynamics is local and not transported. Then, we estimate the entropy transfer related to the spectrum intensity of SRB. Their association reveals the coupling constant value that connects these two parameters and characterizes the strength of wave-particle interactions, namely, the impact of SRB wave activity on SEP thermodynamics. In particular, in the “Measuring the entropic transfer” section, we analyze all the SRB wave activity throughout ~30 min after the trigger time (Figs. 2 and 4). We examine the statistical fits and correlations between the SRB intensities and SEP entropy transfer and verify that they match the expression in Eq. 2.

It is now straightforward to use the analysis procedures developed here to investigate wave-particle interactions across other SEP events. It allows for a systematic investigation on this topic by establishing a consistent characterization of the wave-particle coupling that can reveal a potential variation of the couplings over different types of wave activities and particle populations. In general, (i) we first need to have an abundance of SEPs, characterized by reliable spectra; then (ii) we must detect a systematic and coherent (statistically significant) increase in kappa, (iii) followed by a verification with a decreasing polytropic index ν; (iv) this corresponds to an entropy transfer; and last, (v) we search for the causing SRB or another interacting wave activity.

Further investigations should follow these protocolled lines of research (summarized in Table 1):

**Table 1.** Derivation of coupling constant between wave activity and particle population. Following the paradigm of SRB wave activity affecting SEP thermodynamics, we summarize the five steps for deriving the coupling constant between the wave activity and the affected particle population.

Input	Method	Output
1) Particle spectra	Nonlinear fitting, kappa-tail technique	κ(*t*), *n*(*t*), *T*(*t*)
2) κ(*t*)	Goodness of fit of a constant model	Significant increase in kappa, *d*κ(*t*)/*dt* > 0
3) *n*(*t*) and *T*(*t*)	Evolution of *n*(*t*) and *T*(*t*) in polytropic processes	Polytropic index ν(*t*); validation of κ(*t*) + ν(*t*) ≈ const.
4) *d*κ(*t*)/*dt*	Transport equation of kappa	Entropy transfer, *d*ln(Δ*S*)/*dt*
5) τ∙*d*ln(Δ*S*)/*dt*, *J*/*J*_0_	Relation of entropy transfer & wave intensity	Calculate coupling constant, *k*_wp_

1) At high energies, the kappa distributed flux degenerates to a power-law tail that makes it impossible to be analyzed by standard fitting methods. For this, we first determine whether the examined energy-flux spectrum lies in sufficient high energy, that is, ε ≫(κ − 3/2)*T*, where the linearization approach of the spectrum in log scales can apply. If yes, the thermodynamic parameters can be derived from the kappa-tail technique, i.e., kappa coincides with the spectral index γ = κ, while density and temperature are extracted from analyzing together the spectral index and intercept of the linear fit of spectra on log-log scales; if not, then a typical nonlinear fitting can be performed to determine the thermodynamic parameters involved in the formalism of kappa distribution.

2) Thermodynamic kappa time series includes all the information of entropy transfer. Detecting a coherent (statistically significant) trend of increasing (or decreasing) rate, deviating from an initial nearly constant value (that yet may be characterized by some random fluctuations), measures an entropy transfer to (or from) the particle system, and thus affects its thermodynamics.

3) The same information about entropy transfer can be extracted from the variation of polytropic index. According to the connection of kappa distributions with polytropic processes, the amount that kappa increases corresponds to the amount that the polytropic index decreases, and vice versa. The polytropic index can be determined from various methods, but the most convenient here is to analyze the covariance of density and temperature. Matching the relationship of kappa and polytropic index can be used for validation and improvement of the precision of the kappa time series and for verification of the locality of the coherent variation of kappa.

4) Once the increase (or decrease) in the kappa is determined and validated, it can be used to derive the entropy transfer. For this, the transport equation of kappa must be applied, which can be integrated to give the entropy transfer.

5) The estimated normalized entropy transfer can be linked to the normalized spectral intensity of the observed wave activity. A power-law behavior is expected with the exponent near ~2, while the proportionality constant includes the coupling constant *k*_wp_ of the wave-particle interaction that connects the wave activity with the SEP population, transferring entropy to SEPs and affecting their thermodynamics.

The coupling constant of the wave-particle interactions might be universal for different transient events but characteristic for the involved type of waves and particle populations. Here, we find that this constant is $k_{\text{wp}}≅0.041\pm 0.009$ in the case of the type III SRBs and SEP protons. Further systematic investigation is needed for a consistent characterization of this coupling.

The presented finding of thermodynamic association and entropy transfer between type III wave activity and SEPs allows for the study of the mechanisms behind this thermodynamic connection and a systematic characterization of the coupling of wave-particle interactions by merely using SEP spectra. It also sets the paradigm for further investigations among other types of wave activities and particle populations. These investigations will be crucial in determining the nature and mechanisms interwoven with the coupling of wave-particle interactions.

## MATERIALS AND METHODS

### Observational data

We use data from three instrument suites on board the PSP spacecraft (*58*): FIELDS, IS⊙IS, and SWEAP/SPAN-I for solar wind velocity (*59*) and SWEAP/SPAN-e for electron density (*60*). Energetic proton intensities with particle energy from 10 to 60 MeV are measured by the HET-A of EPI-Hi from the IS⊙IS (*61*) instrument suite. The spectra of these proton intensities are used to derive thermodynamic parameters, e.g., kappa, density, and temperature, which parameterize the kappa distributions and their thermodynamics (*24*, *25*).

Interplanetary magnetic field observations are measured by the magnetometer from the FIELDS instrument suite (*62*), along with radio frequency spectrum (RFS) monitoring. RFS is a two-channel digital receiver, which performs remote sensing observations of radio waves and in situ measurements of electrostatic and electromagnetic fluctuations in the solar wind (*63*). Here, the RFS data are used to determine periods of wave activity in conjunction with nonstationary periods observed in the distributions of energetic proton intensity.

The period of interest is on 16 February 2022 between the hours of 13:00 and 14:00 UTC. This coincides with an SEP event associated with an ICME and its driven shock (*49*, *64*). During this period, PSP has already crossed the interplanetary shock but has not crossed into the ICME and is therefore still within the PUC region (*50*–*52*).

### Thermodynamics of SEPs

The formalism of *D*-dimensional velocity kappa distribution (*9*, *14*), is

$$P_{u}\left(u\right)\sim {\left[1+\frac{1}{\kappa_{0}}\cdot \frac{\varepsilon \left(u\right)}{k_{B}T}\right]}^{-\kappa_{0}-1-\frac{1}{2}D}$$ (4)

with particle kinetic energy with $\varepsilon \left(u\right)=\frac{1}{2}m\cdot {\left(u-u_{b}\right)}^{2}$ , where u and $u_{b}$ are the particle and bulk (mean) velocities, respectively. The distribution is normalized over the velocity space and is parameterized for some thermal temperature *T* and kappa κ_0_. The latter denotes the invariant kappa parameter, a suitable expression thermodynamic kappa when the dimensionality varies. The thermodynamic kappa parameter κ depends on the effective dimensionality or degrees of freedom *D* as

$$\kappa \left(D\right)=\text{const}.+\frac{1}{2}D$$ (5)

where the constant comprises the invariant kappa $\kappa_{0}$ . The physical meaning of the thermodynamic kappa is made clear by its invariant value $\kappa_{0}$ , because this is independent of the degrees of freedom (*9*, *13*, *65*). We use either the notion of the invariant kappa $\kappa_{0}$ or the typical three-dimensional kappa $\kappa \left(3\right)=\kappa_{0}+\frac{3}{2}$.

In the case of energetic protons, the flow speed is negligible, $u_{b}<<u$ , and the kinetic energy directly expresses the particle kinetic energy in the heliospheric reference frame. Then, the kappa distribution of energy and the kappa distributed flux are now simply given by

$$P_{E}\left(\varepsilon \right)\sim {\left(1+\frac{1}{\kappa -\frac{3}{2}}\cdot \frac{\varepsilon }{k_{B}T}\right)}^{-\kappa -1}\;\text{with}\;J\left(\varepsilon \right)=\frac{2}{m^{2}}\cdot n\cdot P_{u}\left(\varepsilon \right)\cdot \varepsilon$$ (6)

The kappa-tail technique is used for finding the thermodynamic values of kappa, temperature, and density, which parameterize the kappa distributed flux of the observed tail of the energy (ε)-flux (*J*) spectra. Fitting the spectra with the linear model $\text{log}J\left(\varepsilon \right)=\text{log}J_{\text{int}}-\gamma \cdot \text{log }\varepsilon$ , we estimate the spectral index, which equals the thermodynamic kappa, $\gamma =\kappa_{0}+\frac{3}{2}=\kappa$ , and the intercept $\text{log}J_{\text{int}}$ or its modified version, $\text{log}{\tilde{J}}_{\text{int}}\equiv \text{log}J_{\text{int}}-\text{log}A\left(\gamma \right)-C$ , where *A*(γ) is a function of spectral index and *C* a constant. Each spectral fit estimates the pair of measurements $\left(\gamma -\frac{1}{2},\text{log}{\tilde{J}}_{\text{int}}\right)$ , and the technique uses these values to find the temperature *T* and density *n* of SEPs (for more details, see the Supplementary Materials) (*24*, *25*).

We derive these parameters by solving $\text{log}{\tilde{J}}_{\text{int}}=\text{log}n+\left(\gamma -\frac{1}{2}\right)\cdot \text{log}T$ (Supplementary Materials); this equation coincides with the polytropic relationship, shown in Eq. 8. However, we have two unknowns for each given pair of measurements $\left(\gamma -\frac{1}{2},\text{log}{\tilde{J}}_{\text{int}}\right)$ . We resolve this by applying the equation for two sequential time intervals. For these intervals, we have two different measurements $\left(\gamma -\frac{1}{2},\text{log}{\tilde{J}}_{\text{int}}\right)$ but the same density and temperature, which are actually characterizing the connection of these two intervals. Using two sequential pair values, e.g., $\left\{\gamma_{L}-\frac{1}{2},{\left(\text{log}{\tilde{J}}_{\text{int}}\right)}_{L}\right\}$ and $\left\{\gamma_{R}-\frac{1}{2},{\left(\text{log}{\tilde{J}}_{\text{int}}\right)}_{R}\right\}$ , we find the density and temperature that characterize the point of connection between two streamline segments

$$\begin{array}{c} \text{log}T_{*}=-\frac{{\left(\text{log}{\tilde{J}}_{\text{int}}\right)}_{R}-{\left(\text{log}{\tilde{J}}_{\text{int}}\right)}_{L}}{\gamma_{R}-\gamma_{L}}\;\text{and}\;\text{log}n_{*}\\ =\frac{\left(\gamma_{R}+\frac{1}{2}\right)\cdot {\left(\text{log}{\tilde{J}}_{\text{int}}\right)}_{L}-\left(\gamma_{L}+\frac{1}{2}\right)\cdot {\left(\text{log}{\tilde{J}}_{\text{int}}\right)}_{R}}{\gamma_{R}-\gamma_{L}} \end{array}$$ (7)

The value of kappa is estimated by the weighted average of the two R and L intervals.

**Fig. 5.**
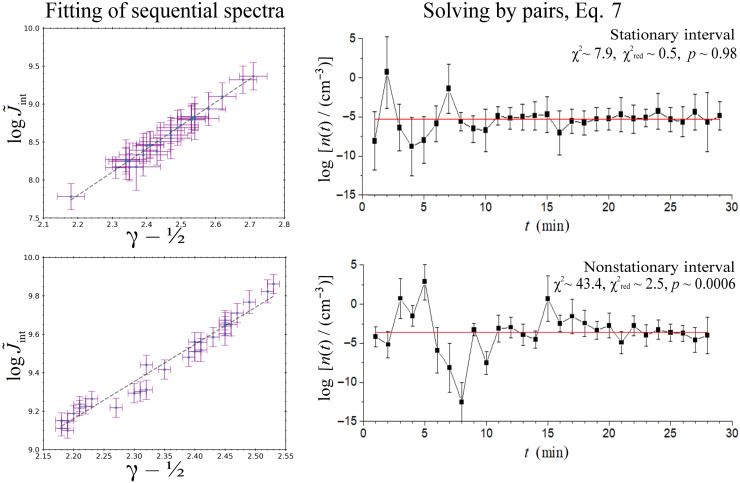
Derivation of thermodynamic parameters using the kappa-tail technique. The panels on the left show the fitting of 30 sequential spectra, while the panels on the right show the case of solving for the density by pairs and the fitting of a constant to these values. The upper panels describe a time period characterized as the “stationary interval” with high statistical significance goodness of fit (*P* value > 0.05), while the lower panels describe a time period characterized as the “nonstationary interval” with low statistical significance of the goodness of fit. (For characterizing the statistical significance, we provide chi-square and *P* values.) Examined periods: for the stationary interval (upper panels), from 01:25 to 01:55 UTC, 16 February 2022; for the nonstationary interval (lower panels), from 13:30 to 14:00 UTC, 16 February 2022.

The kappa-tail technique can be alternatively applied via a linear fit between the spectral index γ and modified intercept $\text{log}{\tilde{J}}_{\text{int}}$ values, taken from a series of a sequential spectra, and then the slope and intercept of this linear regression lead to the temperature and density of SEPs, respectively. Figure 5 (left panels) shows the fitting of 30 sequential spectra, while the respective panels on the right show the case of solving for the density and temperature by pairs, according to Eq. 7.

We examine the stationarity of the values of kappa, temperature, and density by fitting a constant statistical model to the time series of these values (see the Supplementary Materials) (*24*, *25*). Then, the characterization of the goodness of fit will indicate whether the examined values are statistically confident to be characterized as stationary or if they contain nonstationary features, e.g., transient events.

In Fig. 5, the upper panels describe a time period characterized as the “stationary interval,” because the goodness of fit is acceptable, allowing for the derivation of density and temperature. On the contrary, the lower panels describe a time period characterized as the “nonstationary interval,” because the goodness of fit is not acceptable. The reason of the low statistical confidence of this particular example is the fluctuation of density that appears within the first 10 min. This sinusoidal-like fluctuation is an indication that there is a transient event disturbing the thermodynamics of SEPs during this time period. We will be focusing on this same event in the next sections, examining the wave activity, and the transfer of entropy into the thermodynamics of SEPs.

### Polytropic processes of SEPs

During a polytropic process, the thermodynamic parameters evolve as the plasma flows along a streamline, but they remain constrained by a polytropic relationship. This is a power-law expression between thermal pressure *P* and density *n*, $P\propto n^{\gamma_{p}}$ ; given the ideal gas equation of state that also applies for space plasmas, $P=n\;k_{B}T$ , a similar relationship occurs between density *n* and temperature *T*, i.e., $n\propto T^{\nu }$ . Equivalently, the polytropic relationship for logarithms of the thermodynamic parameters is linear

logn−ν⋅logT=const (8)

which is a statistically more robust relationship, as space plasma thermodynamic parameters typically exhibit log-normal variations [e.g., (*66*)]. The primary γ_p_ and secondary ν polytropic indices are related by

$$\gamma_{p}=1+1/\nu \Leftrightarrow \nu =1/\left(\gamma_{p}-1\right)$$ (9)

The constant in Eq. 8 is the polytropic pressure, a thermodynamic parameter that remains invariant along a polytropic process streamline. Nevertheless, the polytropic index is invariant for all streamlines of the process (*67*–*69*).

Polytropes are the particle systems that can be described by the above ideal polytropic behavior at some positional scale and timescale of their flow. They are thermodynamically stationary systems, as they are one-to-one related [e.g., (*14*, *22*)] with the unique kappa distribution that describes their particular stationary thermodynamic state [e.g., (*15*, *69*, *70*)]. Kappa distributions were found to be connected with polytropes through local particle interactions, leading to positional dependence on both the density n(r) and temperature T(r) . Then, kappa distributions have exactly the necessary and sufficient formalism leading to the power law of the polytropic relationship, $n\left(r\right)\propto T{\left(r\right)}^{\nu }$ . The result of this connection is the relationship between thermodynamic kappa κ and polytropic index ν, which is $\kappa_{0}+1+\nu =\frac{1}{2}d_{\Phi }$ , where $d_{\Phi }$ is the degrees of freedom from an attractive potential Φ and is defined by the average particle potential energy <Φ> per temperature [e.g., (*14*, *66*)]; this is generally negligible for SEPs, given that <Φ> is approximately few electron volts [e.g., (*71*, *72*)], while *T* ~ 1 MeV (*24*, *25*). Returning to the standard notation of the three-dimensional kappa, we end up with the kappa-polytropic relationship for SEPs

$$\kappa +\nu -\frac{1}{2}≅0$$ (10)

Estimation of the polytropic index, that is, the exponent of the polytropic power law, *n* ∝ *T*^ν^, can be performed with various methods, which involve plasma dynamics and polytropic processes, such as (i) evolution of thermodynamic parameters along streamlines [e.g., (*68*, *73*–*76*)], (ii) ion-acoustic wave dispersion [e.g., (*14*, *77*)], (iii) Debye length and mean free path [e.g., (*7*, *8*)], (iv) Rankin-Huguenot condition equations over shock discontinuities [e.g., (*78*–*80*)], (v) kappa-polytropic relationship [e.g., (*22*, *69*, *71*, *81*)], and (vi) turbulent energy [e.g., (*82*)].

Here, we estimate the polytropic index via the thermodynamic parameters method (i). In particular, we analyze the density and temperature logarithms by fitting a linear model (Eq. 8) within a subinterval of *L* = 8 data points, corresponding to 8 min [e.g., (*74*, *81*, *83*)]; *L* is the size of the moving window that scans the whole 1-hour interval (from 13.30 to 14.00 UTC, 16 February 2022).

We model the time dependence of both the kappa and polytropic index using a power law, i.e., $\kappa \left(t\right)=\kappa \left(t_{0}\right)+\frac{1}{2}A\cdot {\left(t-t_{0}\right)}^{\alpha }$ and $\nu \left(t\right)=\nu \left(t_{0}\right)-\frac{1}{2}A\cdot {\left(t-t_{0}\right)}^{\alpha }$ , where *A* and α=dlnΔκ/dlnΔt denote the amplitude and scale of the time variation, respectively. Then, the variation of kappa from its stationary value at *t* = *t*_0_, $\Delta \kappa \left(t\right)=\kappa \left(t\right)-\kappa \left(t_{0}\right)$ , leads to transferring of degrees of freedom to SEPs, according to $\Delta D\left(t\right)=2\cdot \Delta \kappa \left(t\right)=A\cdot {\left(t-t_{0}\right)}^{\alpha }$ , and transferring of entropy ΔS(t) according to Eq. 1.

### Measuring the entropic transfer

The entropy defect quantifies the decrease in entropy of a system when correlations are developed among its particles. Classically, the entropy of a system sums the entropies of all the individual system’s parts, e.g., particles. However, this differs for systems with correlations such as space plasmas, and then the total entropy is less than the sum of entropies. The missing entropy, caused by the order induced from the particle correlations, is the entropy defect [for details, see (*10*–*12*, *19*, *21*); see also (*15*, *84*, *85*)]. The entropy defect is important for understanding the foundations of thermodynamics in space plasma physics. Using this concept, ref. (*20*) was able to show that the kappa distributions constitute the fundamental distribution function of velocities (or kinetic energies) in space plasma particle populations.

Here, we use a particular application of the entropy defect that formulates the transport equation of kappa as a function of the entropy transfer (*19*). The equation can be solved to provide the values of kappa along flow streamlines of a kappa-distributed plasma, or conversely, given the kappa as a function of time, κ(*t*), the equation can be used to derive the rate of entropy rate transfer, i.e.

$$\frac{\mathrm{dS}}{\mathrm{dt}}=-\frac{1}{2}S_{\infty }^{2}\cdot \frac{d}{\mathrm{dt}}\left(\frac{1}{\kappa }\right),\text{with}\;S_{\infty }=\text{ln}\frac{T^{\frac{3}{2}}}{n}+\text{const}$$ (11)

where the constant included in $S_{\infty }$ is given by $\frac{3}{2}\text{ln}\left(2k_{B}h^{-2}\pi e^{\frac{5}{3}}\sqrt{m_{e}m_{p}}\right)$ [e.g., (*14**,* *65**,* *86*)].

Having modeled the time dependence of thermodynamic kappa, κ(*t*), we can now use it, in combination with Eq. 1, for determining the entropy transfer to SEPs. We determine the time of “trigger” *t*_0_ at which the wave activity starts transferring entropy into the SEP system, affecting their thermodynamics, and then we integrate Eq. 11 from *t*_0_ to *t* and find, ΔS(t) as given in Eq. 1.

### Measuring the coupling of SRB-SEP interaction

**Fig. 6.**
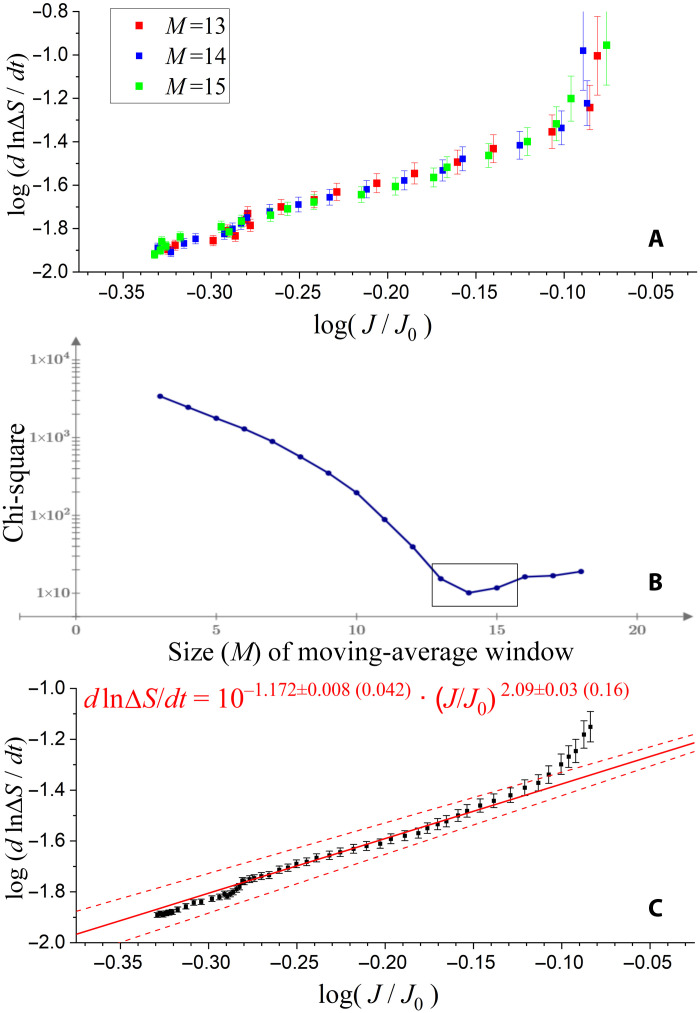
Comparison between the SRB wave activity and entropy transfer. The (logarithms of) entropy rate transfer and spectral intensity are averaged over a moving window of size Δ*t* = *M* (min). Then, they are plotted in (**A**) for the moving window size of 14 (±1) that minimizes the respective chi-square shown in (**B**). The values averaged over all three windows *M* = 13 to 15 are shown in (**C**). The points near the center, from log(*J*/*J*_0_) ~−0.28 to ~−0.14, have a linear trend that can be fitted with a linear model to estimate the values of the wave-particle coupling *k*_wp_ and the intensity exponent *p*.

First, we determine the geometric averages of spectral intensity over the observed intensities [according to (*87*)]. Then, the (logarithmic) values of normalized entropy rate transfer dln(ΔS)/dt and spectral intensity *J*/*J*_0_ are averaged over a moving window of size *t* = *M* (min). Next, their linearity (on a log-log scale) is examined by fitting a linear model and minimizing the respective chi-square. The model is further optimized in terms of the size *M* of the averaging moving window. In Fig. 6A, the entropy rate transfer is plotted against the spectral intensity with a moving window size of 14 (±1) that minimizes the respective chi-square, as shown in Fig. 6B.

Last, we average the values, taken from all the three window sizes *M* = 13 to 15, as follows. First, we mixed the results from all three window sizes, and then we estimate the weighted mean of the values of normalized spectral intensity and entropy rate, estimated per groups of five points. This is plotted in Fig. 6C. The points near the center, from log(*J*/*J*_0_) ~−0.28 to ~−0.14, have a linear trend (a power law or linear in log-log scales); thus, it can be fitted with a linear model to estimate the values of the wave-particle coupling *k*_wp_ and the exponent coupling *p*. A knee appears near log(*J*/*J*_0_) ~−0.28, while the trend appears to be nearly linear in smaller intensities. For larger intensities, the coupling function *f* connecting intensities and entropy transfer expands to higher-order terms and thus deviates from the exponent *p* ~ 2.

## Supplementary Materials

### This PDF file includes:

Supplementary Text S1 and S2 Fig. S1 References
