# Supplementary material for: Entropy transfer from solar radio bursts to energetic particles
Source: Sci Adv. 2025 Nov 26;11(48):eadz7419. doi: 10.1126/sciadv.adz7419 (PMC12652323; doi:10.1126/sciadv.adz7419)
Supplement: Supplementary file 1 — Supplementary Text S1 and S2 Fig. S1 References [file sciadv.adz7419_sm.pdf]

Science Advances
AAAS

Supplementary Materials for

Entropy transfer from solar radio bursts to energetic particles

George Livadiotis *et al.*

Corresponding author: George Livadiotis, glivadiotis@princeton.edu

Sci. Adv. **11**, eadz7419 (2025)
DOI: 10.1126/sciadv.adz7419

This PDF file includes:

Supplementary Text S1 and S2
Fig. S1
References

Supplemental Text 1: Application of Kappa-Tail technique in kappa distributed flux

1.1. When to use the technique for fitting spectra

Observations of energy (ε)–flux (J) spectrum for SEPs typically refer to a power-law tail, i.e., linear on log-log scale (log J vs. log ε). Asymptotic power-law tails is a mathematic characteristic of numerous distribution functions, and thus it is not a unique property of kappa distributions; indeed, they might be connected to concave or convex energy flux spectra in lower-energies. Even though thermodynamics is uniquely connected with kappa distributions, nonthermal processes may be involved in the production of the observed spectrum. Still, the Kappa-Tail technique determines if the observed power-law tail is indeed associated with kappa distributions, and not by any other formulation, and provides a way to estimate the thermodynamic parameters of this distribution. Once the spectrum lies on energy interval within the high-energy limit, set by $\varepsilon >> \kappa_0 k_B T$, then, the spectrum cannot be statistically separated from a linear behavior (on log-log scale), thus there is no statistical confident parabolic term, and the technique can be applied.

1.2. Mathematical expression

The kappa distributed flux is given by

$$J(\varepsilon) = (2\pi^3 m)^{-\frac{1}{2}} \cdot \mathrm{N}(\kappa_0) \cdot n \cdot \left(k_\mathrm{B} T\right)^{-3/2} \cdot \left(1 + \frac{1}{\kappa_0} \cdot \frac{\varepsilon}{k_\mathrm{B} T}\right)^{-\kappa_0 - \frac{5}{2}} \cdot \varepsilon \,, \tag{S1-1a}$$

with

$$\mathrm{N}(\kappa_0) \equiv \kappa_0^{-\frac{3}{2}} \cdot \frac{\Gamma(\kappa_0 + \frac{5}{2})}{\Gamma(\kappa_0 + 1)} \,, \tag{S1-1b}$$

is degenerated to a power-law once the “1” in the base of the distribution in negligible, i.e., when $\varepsilon >> \kappa_0 k_B T$. Then, we have $J(\varepsilon) = J_\mathrm{int} \cdot \varepsilon^{-\gamma}$ or $\log J(\varepsilon) = \log J_\mathrm{int} - \gamma \cdot \log \varepsilon$, where the spectral index equals the thermodynamic kappa,

$$\gamma = \kappa_0 + \tfrac{3}{2} = \kappa \,, \tag{S1-2}$$

while the intercept $\log J_\mathrm{int}$, modified to $\log \tilde{J}_\mathrm{int} \equiv \log J_\mathrm{int} - \log \mathrm{A}(\gamma) - C$, where the involved function is $\mathrm{A}(\gamma) = (\gamma - \frac{3}{2})^{\gamma+1} \cdot \mathrm{N}(\gamma - \frac{3}{2})$, while the constant is $C \equiv 2 - 0.5 \cdot \log\left(2\pi^3 m[\mathrm{keV}]\right) \cong 6.60$, when density is expressed in [cm^{-3}] and temperature in [keV]. Then, the relationship between the modified intercept and the spectral index becomes a linear expression of density and temperature, i.e.,

$$\log \tilde{J}_\mathrm{int} = \log n + (\gamma - \tfrac{1}{2}) \cdot \log T \;. \tag{S1-3}$$

We observe that Eq.(S1-3) reflects exactly the polytropic relationship of Eq.(8).

In *Materials and methods/Thermodynamics of SEPs*, we use Eq.(S1-2) for deriving the values of kappa; we also show how Eq.(S1-2) can be applied in sequential spectra in order to estimate the temperature and density, e.g., (24,25,68).

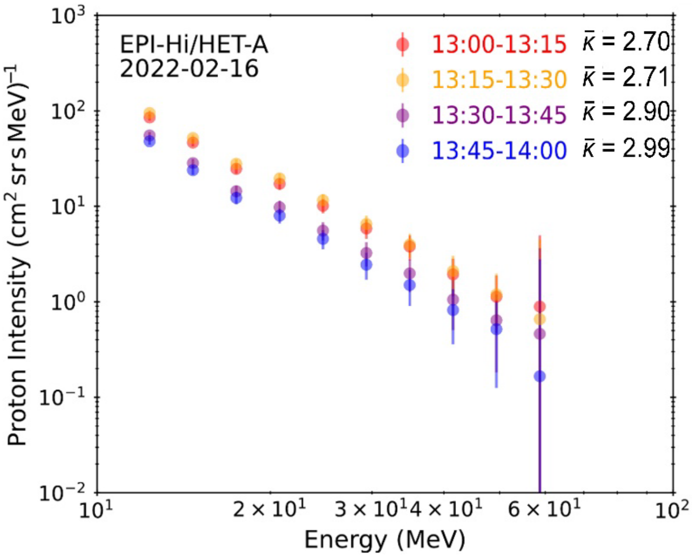

Figure S1. Energy-flux spectrum of SEPs before and after the trigger time where the kappa starts to increase. The spectra are averaged over 15-min window. The corresponding spectral indices (coinciding with the kappa values) are shown.

Supplemental Text 2. Fitting of a mono-parametrical (constant) statistical model

Let the dataset of N pair observations, $\{(x_i, y_i)\}_{i=1}^{N}$, and the respective modelled values $\{f_i\}_{i=1}^{N}$, determined as $f_i \equiv f(x_i)$ from the statistical model $f(x;p) = p$ that best describes the y-data, typically accompanied with the respective uncertainties, $\{y_i \pm \sigma_{yi}\}_{i=1}^{N}$. The fitting involves finding the optimal value of the parameter p that minimizes the normalized sum of the squares of the residuals or chi-square functional (i.e., using the Euclidean norm L_2, (88)), hence,

$$\chi^2 \equiv \sum_{i=1}^{N} \left(\frac{y_i - f_i}{\sigma_{yi}} \right)^2 \ . \qquad \text{(S2-1)}$$

The minimization involves finding the optimal value of the fitting parameter, $p = p^*$, where

$$p^* = \bar{y} = \sum_{i=1}^{N} \frac{y_i}{\sigma_{yi}^{\ 2}} \Big/ \sum_{i=1}^{N} \frac{1}{\sigma_{yi}^{\ 2}} \ . \qquad \text{(S2-2)}$$

In order to derive the fitting error, the chi-square is expanded near its minimum,

$$\chi^2(p) \cong A_0 + A_2 \cdot (p - p^*)^2 + O(p - p^*)^3 . \qquad \text{(S2-3)}$$

The square of the fitting error is proportional to the (reduced) chi-square and inversely proportional to the curvature coefficient A_2 (88,89), i.e.,

$$\delta p^*_{\text{fit}} = \sqrt{\chi^2_{\text{red}} \cdot A_2^{-1}} \;, \tag{S2-4}$$

where $A_0 = \chi^2(p^*)$ is the chi-square minimum, and $\frac{1}{N-1} A_0 = \chi^2_{\text{red}}$ is the reduced chi-square. In our case, the expansion of the chi-square, given by Eq.(B-1), is

$$\chi^2(p) = \sum_{i=1}^{N} \left[\frac{(y_i - p^*) - (p - p^*)}{\sigma_{yi}} \right]^2 = \chi^2(p^*) + \left(\sum_{i=1}^{N} \frac{1}{\sigma_{yi}^2} \right) \cdot (p - p^*)^2 \,, \tag{S2-5}$$

hence, the curvature coefficient and the corresponding fitting error (B-4) become, respectively,

$$A_2 = \sum_{i=1}^{N} \frac{1}{\sigma_{yi}^2} \;,\; \delta p^*_{\text{fit}} = \sqrt{\tfrac{1}{N-1} A_0 \cdot A_2^{-1}} \;. \tag{S2-6}$$

The origin of the fitting error comes from the residuals, i.e., the deviations between the data and model, $\{y_i - f_i\}_{i=1}^N$. The role of the observational uncertainties of the data, $\{\sigma_{yi}\}_{i=1}^N$, in the formulation of this deviation, is simply to express the statistical weights.

The error caused by the observational uncertainties comes from the propagation error. This is a type of error different from the fitting error; it generally differs in its nature, origin, and value. The propagation error is derived from varying the optimal parameter value, expressed in terms of the observation data, $p^* = p^*(\{y_i\}_{i=1}^N)$. In the case of a constant model, we have the simple expression $p^*(\{y_i\}_{i=1}^N) = \bar{y}$. Variation of this function leads to the propagation error,

$$\delta p^*_{\text{prop}} = 1 / \sqrt{\sum_{i=1}^{N} \frac{1}{\sigma_{yi}^2}} \;. \tag{S2-7}$$

We note that the square of the propagation error equals the inverse curvature coefficient, $\delta p^{*2}_{\text{prop}} = A_2^{-1}$. We observe that the chi-square is related to the ratio of the two errors, i.e.,

$$\sqrt{\chi^2_{\text{red}}} = \delta p^*_{\text{fit}} \,/\, \delta p^*_{\text{prop}} \,. \tag{S2-8}$$

Then, the fitting error is given by the standard error form, i.e.,

$$\delta \bar{y}^2 = \tfrac{1}{N-1} \sum_{i=1}^{N} \frac{(y_i - \bar{y})^2}{\sigma_{yi}^2} \Big/ \sum_{i=1}^{N} \frac{1}{\sigma_{yi}^2} \,. \tag{S2-9}$$

Thus, its connection to the reduced chi-square is:

$$\chi^2_{\text{red}} = \tfrac{1}{N-1} \sum_{i=1}^{N} \frac{(y_i - \bar{y})^2}{\sigma_{yi}^2} = \left(\sum_{i=1}^{N} \frac{1}{\sigma_{yi}^2} \right) \cdot \delta \bar{y}^2 \text{, or} \tag{S2-10a}$$

$$\delta p^*_{\text{fit}} = \delta \bar{y} = \sqrt{\chi^2_{\text{red}}} \Big/ \sqrt{\textstyle\sum_{i=1}^{N} \sigma_{yi}^{-2}} \;,\; \delta p^*_{\text{prop}} = 1 \Big/ \sqrt{\textstyle\sum_{i=1}^{N} \sigma_{yi}^{-2}} \;. \tag{S2-10b}$$

Note that in the unbiased version of the above chi-square equations, we must use the effective number of statistical degrees of freedom, instead of N-1, given by (68,90)

$$N_{\text{eq}} = \left(\sum_{i=1}^{N} \sigma_{yi}{}^{-2} \right)^2 / \left(\sum_{i=1}^{N} \sigma_{yi}{}^{-4} \right) - 1 \ . \qquad \text{(S2-11)}$$

Finally, we observe that large deviations between propagation and fitting errors lead to reduced chi-square significantly different than 1, corresponding to low statistical significance of the goodness of the fitting. In the examples of Fig. 5, the “stationary interval” refers to an example where the optimized constant model passes within 1-sigma deviation from all points, while the “nonstationary interval” has a ~10 min period of sinusoidal fluctuation, and the optimized constant misses most of the points within this period.

REFERENCES AND NOTES

1. B. T. Tsurutani, G. S. Lakhina, Some basic concepts of wave-particle interactions in collisionless plasmas. *Rev. Geophys.* **35**, 491–501 (1997).

2. E. Roduner, T. P. J. Krüger, The origin of irreversibility and thermalization in thermodynamic processes. *Phys. Rep.* **944**, 1–43 (2022).

3. P. Zarka, Radio and plasma waves at the outer planets. *Adv. Space Res.* **33**, 2045–2060 (2004).

4. S. D. Baalrud, J. D. Callen, C. C. Hegna, Kinetic theory of instability-enhanced collisional effects. *Phys. Plasmas* **17**, 055704 (2010).

5. H.-J. Li, X.-Z. Zhou, Z.-Y. Liu, S. Wang, Y. Omura, L. Li, C. Yue, Q.-G. Zong, G. Le, C. T. Russell, J. L. Burch, Direct observations of cross-scale wave-particle energy transfer in space plasmas. *Sci. Adv.* **11**, 8227 (2025).

6. J. W. Gibbs, *Elementary Principles in Statistical Mechanics* (Scribner's Sons, 1902).

7. G. Livadiotis, On the generalized formulation of Debye shielding in plasmas. *Phys. Plasmas* **26**, 050701 (2019).

8. G. Livadiotis, Collision frequency and mean free path for plasmas described by kappa distributions. *AIP Adv.* **9**, 10 (2019).

9. G. Livadiotis, D. J. McComas, Invariant kappa distribution in space plasmas out of equilibrium. *Astrophys. J.* **741**, 88 (2011).

10. G. Livadiotis, D. J. McComas, Physical correlations lead to kappa distributions. *Astrophys. J.* **940**, 83 (2022).

11. G. Livadiotis, D. J. McComas, Entropy defect in thermodynamics. *Nat. Sci. Rep.* **13**, 9033 (2023a).

12. G. Livadiotis, D. J. McComas, Entropy defect: Algebra and thermodynamics. *Europhys. Lett.* **144**, 21001 (2023b).

13. G. Livadiotis, Kappa and *q* indices: Dependence on the degrees of freedom. *Entropy* **17**, 2062–2081 (2015).

14. G. Livadiotis, *Kappa Distribution: Theory Applications in Plasmas* (Elsevier, 2017).

15. G. Livadiotis, Thermodynamic origin of kappa distributions. *Europhys. Lett.* **122**, 50001 (2018a).

16. G. Livadiotis, M. I. Desai, L. B. Wilson, Generation of kappa distributions in solar wind at 1 AU. *Astrophys. J.* **853**, 142 (2018).

17. A. Deppman, A. K. Golmankhaneh, E. Megías, R. Pasechnik, From the Boltzmann equation with non-local correlations to a standard non-linear Fokker-Planck equation. *Phys. Lett. B* **839**, 137752 (2023).

18. D. J. McComas, G. Livadiotis, N. V. Sarlis, Correlations and kappa distributions: Numerical experiment and physical understanding. *Entropy* **27**, 375 (2025).

19. G. Livadiotis, D. J. McComas, Transport equation of kappa distributions in the heliosphere. *Astrophys. J.* **954**, 72 (2023c).

20. G. Livadiotis, D. J. McComas, Beyond kappa distributions: Exploiting Tsallis statistical mechanics in space plasmas. *J. Geophys. Res.* **114**, 11105 (2009).

21. G. Livadiotis, D. J. McComas, Thermodynamic definitions of temperature and kappa and introduction of the entropy defect. *Entropy* **23**, 1683 (2021).

22. G. Livadiotis, On the origin of polytropic behavior in space and astrophysical plasmas. *Astrophys. J.* **874**, 10 (2019).

23. G. Livadiotis, D. J. McComas, Measure of the departure of the q-metastable stationary states from equilibrium. *Phys Scr* **82**, 035003 (2010).

24. G. Livadiotis, A.T. Cummings, M. E. Cuesta, R. Bandyopadhyay, H. A. Farooki, L. Y. Khoo, D. J. McComas, J. S. Rankin, T. Sharma, M. M. Shen, C. M. S. Cohen, G. D. Muro, Z. Xu, Kappa-tail technique: Modeling and application to solar energetic particles observed by Parker Solar Probe. *Astrophys. J.* **973**, 6 (2024).

25. M. E. Cuesta, A. T. Cummings, G. Livadiotis, D. J. McComas, C. M. S. Cohen, L. Y. Khoo, T. Sharma, M. M. Shen, R. Bandyopadhyay, J. S. Rankin, J. R. Szalay, H. A. Farooki, Z. Xu, G. D. Muro, M. L. Stevens, S. D. Bale, Observations of kappa distributions in solar energetic protons and derived thermodynamic properties. *Astrophys. J.* **973**, 76 (2024).

26. W. I. Axford, E. Leer, G. Skadron, "The acceleration of cosmic rays by shock waves," in *15th International Cosmic Ray Conference* (Dept. of Cosmic Rays, Central Research Institute for Physics of the Hungarian Academy of Sciences, 1977), vol. **11**, p. 132.

27. A. R. Bell, The acceleration of cosmic rays in shock fronts—I. *Month. Not. Royal Astron. Soc.* **182**, 147–156 (1978).

28. R. D. Blandford, J. P. Ostriker, Particle acceleration by astrophysical shocks. *Astrophys. J. Let.* **221**, L29–32 (1978).

29. J. T. Gosling, J. R. Asbridge, S. J. Bame, W. C. Feldman, Ion acceleration at the earth's bow shock: A review of observations in the upstream region. *AIP Conf. Proc.* **56**, 81–99 (1979).

30. M. A. Lee, Particle acceleration and transport at CME-driven shocks. *Geophys. Monograph Ser.* **99**, 227 (1997).

31. G. M. Mason, G. Gloeckler, Power law distributions of suprathermal ions in the quiet solar wind. *Space Sci. Rev.* **172**, 241–251 (2012).

32. I. P. Wild, S. F. Smerd, Radio bursts from the solar corona. *Ann. Rev. Astron. Astrophys.* **10**, 159–196 (1972).

33. D. B. Melrose, The emission mechanisms for solar radio bursts. *Space Sci. Rev.* **26**, 3–38 (1980).

34. G. A. Dulk, Type III solar radio bursts at long wavelengths. *Geophys. Monogr. Ser.* **119**, 115 (2000).

35. N. Gopalswamy, Recent advances in the long-wavelength radio physics of the Sun. *Planet. Space Sci.* **52**, 1399–1413 (2004).

36. H. A. S. Reid, H. Ratcliffe, A review of solar type III radio bursts. *Res. Astron. Astrophys.* **14**, 773–804 (2014).

37. D. V. Reames, The two sources of solar energetic particles. *Space Sci. Rev.* **175**, 53–92 (2013).

38. N. Dresing, R. Gómez-Herrero, B. Heber, A. Klassen, M. Temmer, A. Veronig, Long-lasting injection of solar energetic electrons into the heliosphere. *Astron. Astrophys.* **613**, A21 (2018).

39. L. Wang, R. P. Lin, S. Krucker, G. M. Mason, A statistical study of solar electron events over one solar cycle. *Astrophys. J.* **759**, 69 (2012).

40. R. E. Ergun, D. Larson, R. P. Lin, J. P. McFadden, C. W. Carlson, K. A. Anderson, L. Muschietti, M. McCarthy, G. K. Parks, H. Reme, J. M. Bosqued, C. D'Uston, T. R. Sanderson, K. P. Wenzel, M. Kaiser, R. P. Lepping, S. D. Bale, P. Kellogg, J. L. Bougeret, Wind spacecraft observations of solar impulsive electron events associated with solar type III radio bursts. *Astrophys. J.* **503**, 435–445 (1998).

41. M. Pulupa, S. D. Bale, S. T. Badman, J. W. Bonnell, A. W. Case, T. Dudok de Wit, K. Goetz, P. R. Harvey, A. M. Hegedus, J. C. Kasper, K. E. Korreck, V. Krasnoselskikh, D. Larson, A. Lecacheux, R. Livi, R. J. MacDowall, M. Maksimovic, D. M. Malaspina, J. C. M. Oliveros, N. Meyer-Vernet, M. Moncuquet, M. Stevens, P. Whittlesey, Statistics and polarization of Type III radio bursts observed in the inner heliosphere. *Astrophys. J. Suppl. Ser.* **246**, 49 (2020).

42. J. G. Mitchell, E. R. Christian, G. A. de Nolfo, C. M. S. Cohen, M. E. Hill, A. Kouloumvakos, A. W. Labrador, R. A. Leske, D. J. McComas, R. L. McNutt, Jr., D. G. Mitchell, M. Shen, N. A. Schwadron, M. E. Wiedenbeck, S. D. Bale, M. Pulupa, Delay of near-relativistic electrons with respect to type III radio bursts throughout the inner heliosphere. *Astrophys. J.* **980**, 96 (2025).

43. S. W. Kahler, E. W. Cliver, H. V. Cane, R. E. McGuire, R. G. Stone, N. R. Sheeley Jr, Solar filament eruptions and energetic particle events. *Astrophys. J.* **302**, 504 (1986).

44. R. J. MacDowall, A. Lara, P. K. Manoharan, N. V. Nitta, A. M. Rosas, J. L. Bougeret, Long-duration hectometric type III radio bursts and their association with solar energetic particle (SEP) events. *Geophys. Res. Let.* **30**, 8018 (2003).

45. H. V. Cane, R. G. Stone, Type II solar radio bursts, interplanetary shocks, and energetic particle events. *Astrophys. J.* **282**, 339–344 (1984).

46. V. Zhdankin, Generalized entropy production in collisionless plasma flows and turbulence. *Phys. Rev. X* **12**, 031011 (2022).

47. G. W. Collins II, *The Fundamentals of Stellar Astrophysics* (Freeman, 1989), chapter 15.

48. M. E. Cuesta, G. Livadiotis, D. J. McComas, Transfer of entropy between the magnetic field and solar energetic particles during an interplanetary coronal mass ejection. *Astrophys. J. Lett.* **984**, L50 (2025).

49. T. Thapa, Y. Yan, Time profile study of type III solar radio bursts using Parker Solar Probe. *Astrophys. J.* **972**, 2 (2024).

50. E. Palmerio, F. Carcaboso, L. Y. Khoo, T. M. Salman, B. Sánchez-Cano, B. J. Lynch, Y. J. Rivera, S. Pal, T. Nieves-Chinchilla, A. J. Weiss, D. Lario, J. Z. D. Mieth, D. Heyner, M. L. Stevens, O. M. Romeo, A. N. Zhukov, L. Rodriguez, C. O. Lee, C. M. S. Cohen, L. Rodríguez-García, P. L. Whittlesey, N. Dresing, P. Oleynik, I. C. Jebaraj, D. Fischer, D. Schmid, I. Richter, H.-U. Auster, F. Fraschetti, M. Mierla, On the mesoscale structure of coronal mass ejections at Mercury's orbit: Bepicolombo and Parker Solar Probe observations. *Astrophys. J.* **963**, 108 (2024).

51. I. Das, M. Opher, R. Evans, C. Loesch, T. I. Gombosi, Evolution of piled-up compressions in modeled coronal mass ejection sheaths and the resulting sheath structures. *Astrophys. J.* **729**, 112 (2011).

52. J. Farrugia, N. V. Erkaev, U. Taubenschuss, V. A. Shaidurov, C. W. Smith, H. K. Biernat, A slow mode transition region adjoining the front boundary of a magnetic cloud as a relic of a convected solar wind feature: Observations and MHD simulation. *J. Geophys. Res.* **113**, A00B01 (2008).

53. H. A. S. Reid, A review of recent solar type III imaging spectroscopy. *Front. Astron. Space Sci.* **7**, 2020 (2020).

54. C. M. S. Cohen, E. R. Christian, A. C. Cummings, A. J. Davis, M. I. Desai, G. A. de Nolfo, J. Giacalone, M. E. Hill, C. J. Joyce, A. W. Labrador, R. A. Leske, W. H. Matthaeus, D. J. McComas, R. L. McNutt Jr., R. A. Mewaldt, D. G. Mitchell, J. G. Mitchell, J. S. Rankin, E. C. Roelof, N. A. Schwadron, E. C. Stone, J. R. Szalay, M. E. Wiedenbeck, A. Vourlidas, S. D. Bale, M. Pulupa, R. J. MacDowall, PSP/IS⨀IS observations of the 29 November 2020 solar energetic particle event. *Astron. Astrophys.* **656**, A29 (2021).

55. B. Ma, L. Chen, D. Wu, Z. Ning, M. Pulupa, S. D. Bale, Type IV-like solar radio burst consisting of a series of short-time bursts observed by PSP. *Astrophys. J.* **979**, 22 (2025).

56. M. Nakata, T.-H. Watanabe, H. Sugama, Nonlinear entropy transfer via zonal flows in gyrokinetic plasma turbulence. *Phys. Plasmas* **19**, 022303, 2012.

57. S. Du, G. P. Zank, X. Li, F. Guo, Energy dissipation and entropy in collisionless plasma. *Phys. Rev. E* **101**, 033208 (2020).

58. N. J. Fox, M. C. Velli, S. D. Bale, R. Decker, A. Driesman, R. A. Howard, J. C. Kasper, J. Kinnison, M. Kusterer, D. Lario, M. K. Lockwood, D. J. McComas, N. E. Raouafi, A. Szabo, The solar probe plus mission: Humanity's first visit to our star. *Space Sci. Rev.* **204**, 7–48 (2016)

59. R. Livi, D. E. Larson, J. C. Kasper, R. Abiad, A. W. Case, K. G. Klein, D. W. Curtis, G. Dalton, M. Stevens, K. E. Korreck, G. Ho, M. Robinson, C. Tiu, P. L. Whittlesey, J. L. Verniero, J. Halekas, J. McFadden, M. Marckwordt, A. Slagle, M. Abatcha, A. Rahmati, M. D. McManus, The Solar Probe ANalyzer—Ions on the Parker Solar Probe. *Astrophys. J.* **938**, 138 (2022).

60. P. L. Whittlesey, D. E. Larson, J. C. Kasper, J. Halekas, M. Abatcha, R. Abiad, M. Berthomier, A. W. Case, J. Chen, D. W. Curtis, G. Dalton, K. G. Klein, K. E. Korreck, R. Livi, M. Ludlam, M. Marckwordt, A. Rahmati, M. Robinson, A. Slagle, M. L. Stevens, C. Tiu, J. L. Verniero, The Solar Probe ANalyzers—Electrons on the Parker Solar Probe. *Astrophys. J. Suppl. Ser.* **246**, 74 (2020).

61. D. J. McComas, N. Alexander, N. Angold, S. Bale, C. Beebe, B. Birdwell, M. Boyle, J. M. Burgum, J. A. Burnham, E. R. Christian, W. R. Cook, S. A. Cooper, A. C. Cummings, A. J. Davis, M. I. Desai, J. Dickinson, G. Dirks, D. H. Do, N. Fox, J. Giacalone, R. E. Gold, R. S. Gurnee, J. R. Hayes, M. E. Hill, J. C. Kasper, B. Kecman, J. Klemic, S. M. Krimigis, A. W. Labrador, R. S. Layman, R. A. Leske, S. Livi, W. H. Matthaeus, R. L. McNutt Jr, R. A. Mewaldt, D. G. Mitchell, K. S. Nelson, C. Parker, J. S. Rankin, E. C. Roelof, N. A. Schwadron, H. Seifert, S. Shuman, M. R. Stokes, E. C. Stone, J. D. Vandegriff, M. Velli, T. T. von Rosenvinge, S. E. Weidner, M. E. Wiedenbeck, P. Wilson IV, Integrated Science Investigation of the Sun (ISIS): Design of the energetic particle investigation. *Space Sci. Rev.* **204**, 187–256 (2016).

62. S. D. Bale, K. Goetz, P. R. Harvey, P. Turin, J. W. Bonnell, T. Dudok de Wit, R. E. Ergun, R. J. MacDowall, M. Pulupa, M. Andre, M. Bolton, J.-L. Bougeret, T. A. Bowen, D. Burgess, C. A. Cattell, B. D. G. Chandran, C. C. Chaston, C. H. K. Chen, M. K. Choi, J. E. Connerney, S. Cranmer, M. Diaz-Aguado, W. Donakowski, J. F. Drake, W. M. Farrell, P. Fergeau, J. Fermin, J. Fischer, N. Fox, D. Glaser, M. Goldstein, D. Gordon, E. Hanson, S. E. Harris, L. M. Hayes, J. J. Hinze, J. V. Hollweg, T. S. Horbury, R. A. Howard, V. Hoxie, G. Jannet, M. Karlsson, J. C. Kasper, P. J. Kellogg, M. Kien, J. A. Klimchuk, V. V. Krasnoselskikh, S. Krucker, J. J. Lynch, M. Maksimovic, D. M. Malaspina, S. Marker, P. Martin, J. Martinez-Oliveros, J. McCauley, D. J. McComas, T. McDonald, N. Meyer-Vernet, M. Moncuquet, S. J. Monson, F. S. Mozer, S. D. Murphy, J. Odom, R. Oliverson, J. Olson, E. N. Parker, D. Pankow, T. Phan, E. Quataert, T. Quinn, S. W. Ruplin, C. Salem, D. Seitz, D. A. Sheppard, A. Siy, K. Stevens, D. Summers, A. Szabo, M. Timofeeva, A. Vaivads, M. Velli, A. Yehle, D. Werthimer, J. R. Wygant, The FIELDS instrument suite for Solar Probe Plus. Measuring the coronal plasma and magnetic field, plasma waves and turbulence, and radio signatures of solar transients. *Space Sci. Rev.* **204**, 49–82 (2016).

63. M. Pulupa, S. D. Bale, J. W. Bonnell, T. A. Bowen, N. Carruth, K. Goetz, D. Gordon, P. R. Harvey, M. Maksimovic, J. C. Martínez-Oliveros, M. Moncuquet, P. Saint-Hilaire, D. Seitz, D. Sundkvist, The Solar Probe Plus radio frequency spectrometer: Measurement requirements, analog design, and digital signal processing. *J. Geophys. Res.* **122**, 2836–2854 (2017).

64. L. Y. Khoo, B. Sánchez-Cano, C. O. Lee, L. Rodríguez-García, A. Kouloumvakos, E. Palmerio, F. Carcaboso, D. Lario, N. Dresing, C. M. S. Cohen, D. J. McComas, B. J. Lynch, F. Fraschetti, I. C. Jebaraj, J. G. Mitchell, T. Nieves-Chinchilla, V. Krupar, D. Pacheco, J. Giacalone, H.-U. Auster, J. Benkhoff, X. Bonnin, E. R. Christian, B. Ehresmann, A. Fedeli, D. Fischer, D. Heyner, M. Holmström, R. A. Leske, M. Maksimovic, J. Z. D. Mieth, P. Oleynik, M. Pinto, I. Richter, J. Rodríguez-Pacheco, N. A. Schwadron, D. Schmid, D. Telloni, A. Vecchio, M. E. Wiedenbeck, Multispacecraft observations of a widespread solar energetic particle event on 2022 February 15–16. *Astrophys. J.* **963**, 107 (2024).

65. G. Livadiotis, Lagrangian temperature: Derivation and physical meaning for systems described by kappa distributions. *Entropy* **16**, 4290–4308 (2014).

66. I. S. Veselovskya, K. B. Kaportseva, Role of averaging in statistical analysis of solar wind data from the DSCOVR spacecraft for the first year of operation. *Geomagn. Aeron.* **59**, 257–264 (2019).

67. G. Livadiotis, Superposition of polytropes in the inner heliosheath. *Astrophys. J. Suppl. Ser.* **223**, 13 (2016).

68. G. Livadiotis, D. J. McComas, H. O. Funsten, N. A. Schwadron, J. R. Szalay, E. Zirnstein, Thermodynamics of the inner heliosheath. *Astrophys. J. Suppl. Ser.* **262**, 53 (2022).

69. G. Livadiotis, D. J. McComas, What defines stationarity in space plasmas. *Astrophys. J.* **982**, 169 (2025).

70. S. Abe, General pseudoadditivity of composable entropy prescribed by the existence of equilibrium. *Phys. Rev. E* **63**, 061105 (2001).

71. G. Livadiotis, Using kappa distributions to identify the potential energy. *J. Geophys. Res.* **123**, 1050–1060 (2018).

72. C. Lacombe, C. Salem, A. Mangeney, D. Hubert, C. Perche, J.-L. Bougeret, P. J. Kellogg, J.-M. Bosqued, Evidence for the interplanetary electric potential? WIND observations of electrostatic fluctuations. *Ann. Geophys.* **20**, 609–618 (2002).

73. G. Livadiotis, Long-term independence of solar wind polytropic index on plasma flow speed. *Entropy* **20**, 799 (2018).

74. G. Nicolaou, G. Livadiotis, R. T. Wicks, D. Verscharen, B. A. Maruca, Polytropic behavior of solar wind protons observed by Parker Solar Probe. *Astrophys. J.* **901**, 26 (2020).

75. M. A. Dayeh, G. Livadiotis, Polytropic behavior in the structures of interplanetary coronal mass ejections. *Astrophys. J Let.* **941**, L26 (2022).

76. C. Katsavrias, G. Nicolaou, G. Livadiotis, A. Vourlidas, L. B. Wilson III, I. Sandberg, Dependence of the polytropic behaviour of solar wind protons on temperature anisotropy and plasma β near L1. *Astron. & Astrophys.* **691**, L11 (2024).

77. P. H. Yoon, *Classical Kinetic Theory of Weakly Turbulent Nonlinear Plasma Processes* (Cambridge Univ. Press, Cambridge, 2019).

78. M. Tatrallyay, C. T. Russell, J. G. Luhmann, A. Barnes, J. G. Mihalov, On the proper Mach number and ratio of specific heats for modeling the Venus bow shock. *J. Geophys. Res.* **89**, 7381–7392 (1984).

79. D. Winterhalter, M. G. Kivelson, R. J. Walker, C. T. Russell, The MHD Rankine-Hugoniot jump conditions and the terrestrial bow shock: A statistical comparison. *Adv. Space Res.* **4**, 287–292 (1984)

80. G. Livadiotis, Shock strength in space and astrophysical plasmas. *Astrophys. J.* **809**, 111 (2015b).

81. G. Nicolaou, G. Livadiotis, Long-term correlations of polytropic indices with kappa distributions in solar wind plasma near 1 au. *Astrophys. J.* **884**, 52 (2019).

82. G. Livadiotis, D. J. McComas, Connection between polytropic index and heating. *Astrophys. J.* **956**, 88 (2023).

83. M. Kartalev, M. Dryer, K. Grigorov, E. Stoimenova, Solar wind polytropic index estimates based on single spacecraft plasma and interplanetary magnetic field measurements. *J. Geophys. Res.* **111**, A10107 (2006).

84. A. Enciso, P. Tempesta, Uniqueness and characterization theorems for generalized entropies. *J. Stat. Mech.* **2017**, 123101 (2017).

85. K. Ourabah, Superstatistics from a dynamical perspective: Entropy and relaxation. *Phys. Rev. E* **109**, 014127 (2024).

86. G. Livadiotis, D. J. McComas, Evidence of large-scale quantization in space plasmas. *Entropy* **15**, 1118–1134 (2013).

87. M. E. Cuesta, L. Y. Khoo, G. Livadiotis, M. M. Shen, J. R. Szalay, D. J. McComas, J. S. Rankin, R. Bandyopadhyay, H. A. Farooki, J. T. Niehof, C. M. S. Cohen, R. A. Leske, Z. Xu, E. R. Christian, M. I. Desai, M. A. Dayeh, Comparing methods for calculating solar energetic particle intensities: Rebinning versus spectral binning. *Astrophys. J.* **980**, 235 (2025).

88. G. Livadiotis, Approach to general methods for fitting and their sensitivity, *Phys. A* **375**, 518–536 (2007).

89. G. Livadiotis, Chi-p distribution: Characterization of the goodness of the fitting using Lp norms, *J. Stat. Distr. Appl.* **1**, 1–14 (2014).

90. P. R. Bevington, *Data Reduction and Error Analysis for the Physical Sciences* (McGraw-Hill, 1969), pp. 336.
